# The impact of dose calculation algorithms on partial and whole breast radiation treatment plans

**DOI:** 10.1186/1748-717X-5-120

**Published:** 2010-12-16

**Authors:** Parminder S Basran, Sergei Zavgorodni, Tanya Berrang, Ivo A Olivotto, Wayne Beckham

**Affiliations:** 1 Department of Medical Physics, BC Cancer Agency--Vancouver Island Centre, Victoria, British Columbia, Canada; 2 Department of Physics and Astronomy, University of Victoria, Victoria, British Columbia, Canada; 3 Department of Radiation Oncology, BC Cancer Agency,Vancouver Island Centre, Victoria, British Columbia, Canada; 4 Department of Surgery, University of British Columbia, Vancouver, British Columbia, Canada

## Abstract

**Background:**

This paper compares the calculated dose to target and normal tissues when using pencil beam (PBC), superposition/convolution (AAA) and Monte Carlo (MC) algorithms for whole breast (WBI) and accelerated partial breast irradiation (APBI) treatment plans.

**Methods:**

Plans for 10 patients who met all dosimetry constraints on a prospective APBI protocol when using PBC calculations were recomputed with AAA and MC, keeping the monitor units and beam angles fixed. Similar calculations were performed for WBI plans on the same patients. Doses to target and normal tissue volumes were tested for significance using the paired Student's t-test.

**Results:**

For WBI plans the average dose to target volumes when using PBC calculations was not significantly different than AAA calculations, the average PBC dose to the ipsilateral breast was 10.5% higher than the AAA calculations and the average MC dose to the ipsilateral breast was 11.8% lower than the PBC calculations. For ABPI plans there were no differences in dose to the planning target volume, ipsilateral breast, heart, ipsilateral lung, or contra-lateral lung. Although not significant, the maximum PBC dose to the contra-lateral breast was 1.9% higher than AAA and the PBC dose to the clinical target volume was 2.1% higher than AAA. When WBI technique is switched to APBI, there was significant reduction in dose to the ipsilateral breast when using PBC, a significant reduction in dose to the ipsilateral lung when using AAA, and a significant reduction in dose to the ipsilateral breast and lung and contra-lateral lung when using MC.

**Conclusions:**

There is very good agreement between PBC, AAA and MC for all target and most normal tissues when treating with APBI and WBI and most of the differences in doses to target and normal tissues are not clinically significant. However, a commonly used dosimetry constraint, as recommended by the ASTRO consensus document for APBI, that no point in the contra-lateral breast volume should receive >3% of the prescribed dose needs to be relaxed to >5%.

## Background

For early stage breast cancer, whole breast irradiation (WBI) is used extensively to minimize the risk of ipsilateral breast cancer recurrence after breast conserving surgery. Over the last decade, there has been increased interest in the use of accelerated partial breast irradiation (APBI) as opposed to WBI [1]. The use of APBI offers fewer fractions and lower dose to uninvolved regions of the breast. A number of clinical trials comparing WBI with various methods of APBI treatments are ongoing [2], however mature randomized data on the efficacy and toxicity of APBI compared to standard WBI will not be available for a number of years.

Publications supporting the dosimetric advantages of using APBI as an alternative to WBI have mainly focused on intra-cavitary brachytherapy, interstitial brachytherapy or intra-operative radiation therapy [3-6]. A common method of delivering APBI in ongoing randomized trials is linac-based, 3-dimensional conformal external beam radiation therapy (3DCRT) employing the same widely-available technology, staff, and treatment planning systems as WBI [7].

Given the potential importance of linear accelerator based delivery of APBI, the influence of dose calculation algorithms on trial eligibility and interpretation of risks to normal tissues is relevant. The impact of scatter corrections with WBI techniques comparing pencil beam convolution (PBC), the analytic anisotropic algorithm (AAA), and Monte Carlo (MC) calculations has been previously described [8], with several articles discussing the benefits of using AAA over PBC [9,10]. However, there are no studies that examine the accuracy of the dose to target and normal tissues for 3DCRT APBI techniques. The accuracy of the calculated dose in regions well outside the irradiated volume is particularly important when trying to ascertain the risk of secondary cancer or normal tissue toxicity [11]. Obtaining a better understanding of the potential increase, or decrease, in dose to target and normal tissues could facilitate a better understanding of the risks associated with APBI treatment strategies. This is a report of the consequences of changing dose calculation algorithms on doses to target volumes and important normal tissues during whole breast and partial breast irradiation.

## Methods

### Treatment planning

We retrospectively examined plans for 10 consecutive patients enrolled in a prospective APBI trial who met all the dosimetry constraints of the protocol when using simplified pencil beam calculations [12]. All plans were initially calculated with a pencil beam convolution (PBC) algorithm with Batho inhomogeneity corrections using the Eclipse Treatment Planning System (Version 8.617, Varian Medical Systems, Palo Alto, USA) [13]. Plans were then recomputed (keeping the monitor units, beam weights and angles fixed) within Eclipse using AAA. All calculations were performed on 2.5 mm dose grid.

The WBI prescription was 42.5 Gy in 16 fractions, normalized to a point mid-plane in the breast tissue and to be delivered through a segmented MLC delivery with 6 MV photon beams.

**Figure 1:**
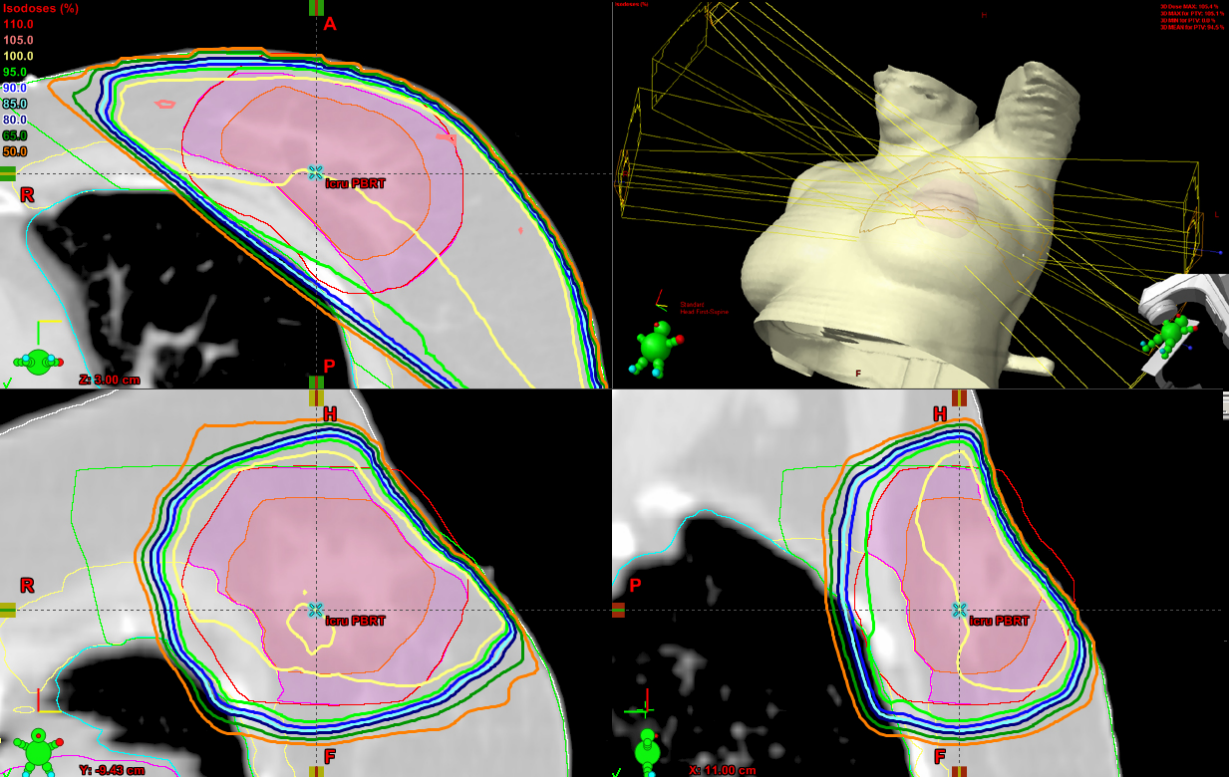
**Transaxial (upper left), coronal (lower left), sagittal (lower right), and three dimensional rendering of a partial breast plan computed with the pencil beam algorithm**. The dose escalation volume (DEV), shown in purple, is a 5 millimeter expansion of the clinical target volume, shown in pink, but excludes the chest wall.

The partial breast technique employed four non-coplanar 6 MV beams that avoided direct beams into the ipsilateral lung [14]. The planning target volume (PTV) for the APBI plans was the seroma (the primary surgical site density on a planning CT scan) plus a 1 cm expansion, excluding chest wall and 0.5 cm from the skin, to form the clinical target volume (CTV) and a further 1 cm 3-dimensional expansion to form the PTV. A dose-evaluation volume (DEV) was defined as the portion of the PTV that excluded the chest wall and 0.5 cm from the skin [14]. In addition to defining these target structures, the ipsilateral breast, ipsilateral lung, heart, contra-lateral lung and contra-lateral breast were contoured (see Figure 1).

The APBI prescription was 38.5 Gy in 10 fractions normalized to a point within the target volume. The planning guidelines for APBI patients follow those articulated in the American Society of Therapeutic Radiology and Oncology (ASTRO) consensus document [1].

### Monte Carlo Verification

WBI and APBI treatment plans were recomputed with the Vancouver Island Monte Carlo (VIMC) system [15,16]. The system provides a platform for Monte Carlo verification of the treatment plans generated by a TPS and exported in DICOM format.

The main "calculation engines" within the system are BEAMnrc for modelling particle fluence and DOSXYZnrc for modelling the dose deposition within the patient [17]. The beam model for Varian 21EX treatment machine was used in this study. The model utilises a two-stage approach in calculating the dose where in the "first stage" all non-variable linac components are modelled and the particle fluence is stored in the phase space file. Then, in the "second stage" the phase space file is used in subsequent calculations as a radiation source for transporting the fluence through the patient phantom. Standard energy cut-off values were AP = PCUT = 0.01 MeV and AE = ECUT = 0.700 MeV, where AP and AE are the low energy thresholds for the production of secondary bremsstrahlung photons and knock-on electrons and PCUT and ECUT are the global cut-off energies for photon and electron transport used during electron and photon transport. In addition, "azimuthal particle redistribution" has been used to substantially reduce phase space latent variance [18,19]. The model has been tuned and verified (except the build-up and penumbra regions) demonstrating dose agreement with the measured open field dose profiles within 1% for the field sizes within the range of 4 × 4 to 40 × 40 cm^2 ^[11]. This excluded build-up and penumbra regions where the dose differences were higher, as expected, but still agreed to within 2% or within a 2 mm distance. Modelling of IMRT and RapidArc, as well as fixed-aperture fields' delivery has been performed with the dynamic multi-leaf collimators (dMLC) model by Siebers et al. and verified in our previous publications [11,20,21].

As most of the treatment fields used in the current study utilise the Varian implementation of collimator-controlled wedging, or enhanced dynamic wedges (EDWs), it is important that the dose from such fields is calculated correctly. Radiation transport through the moving jaw of EDWs is modelled in VIMC system using the method developed by Verhaegen and Liu [22]. Each particle is transported through the dynamic jaw with its position sampled from a probability density function that describes jaw motion. Then, the particle is transported through the physical jaw in its sampled position. This method naturally models the radiation transmitted through the dynamic jaw towards the patient as well as radiation backscattered from the jaw into the linac monitor chamber. The latter is essential for correct absolute dose calculation implemented in the VIMC linac model [23]. Verhaegen and Liu demonstrated excellent agreement of this EDW model with measured data. Our implementation of this model has been verified against the EDW commissioning measurements collected in our department. The measurements were done using Scanditronix Wellhofer CA24 ionization chamber array with IC-10 ionization chambers that have effective volume of 0.13 cm^3^. Examples of this verification for Monte Carlo as well as PBC and AAA calculations that include 10 × 10 and 20 × 20 cm^2 ^fields with 60° wedge are shown in the Results section.

MC simulations of the treatment plans presented in this study were performed on 2.5 mm dose grid with less than 1% statistical uncertainty at the DEV.

### Statistical Analysis

Volumetric and dosimetric statistics as defined in Table 1 were recorded from each of the patient's 6 plans (WBI-PBC, WBI-AAA, WBI-MC, APBI-PBC, APBI-AAA, and APBI-MC). To determine whether there is a difference to these volumes, the mean percentage differences in doses or volumes receiving a specific dose were tested using the paired Student's t-test computed in Microsoft Excel (Microsoft, Redmond WA). For a significance level of p = 0.05, the adjusted significance level with Bonferroni corrections for the 8 different tissues analyzed in this study is p = 0.006 [24].

**Table 1:** Target and normal tissue dosimetric definitions and the average volumes for 10 patients in this study.

Target & Normal Tissue	Average Volume [cm^3^]	Statistic Recorded
Planning Target Volume (PTV)	215.0	Relative volume covered by 95% of the prescription dose
Dose Evaluation Volume (DEV)	149.3	Relative volume covered by 95% of the prescription dose
Ipsilateral Breast (IPS-BR)	1094.5	Relative volume covered by 95% of the prescription dose
Ipsilateral Lung (IPS-LUNG)	1368.1	Relative volume receiving 10% of the prescription dose
Heart	537.4	Percent of prescription dose delivered to 10% of the volume
Contra-lateral lung (CON-LUNG)	1182.0	Percent of prescription dose delivered to 5% of the volume
Contra-lateral breast (CON-BR)	525.2	Maximum point dose as a percent of the prescription dose

## Results

### Verification of MC, AAA and PBC dose calculations for EDW fields

**Figure 2:**
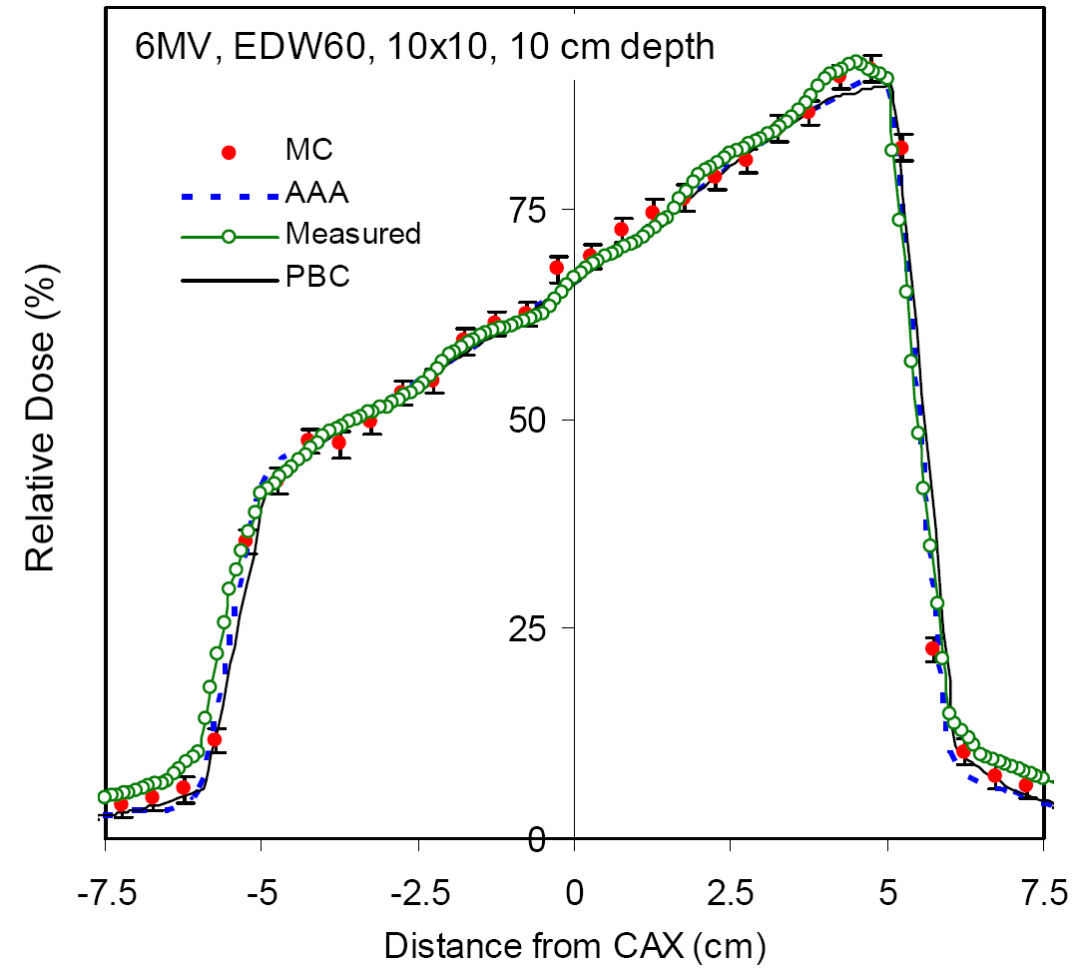
**Dose profile of a 10 × 10 cm^2 ^field at a depth of 10 cm in water for a 60° enhanced dynamic wedge measured with ionisation chamber array (Measured), calculated by Monte Carlo method (MC), as well as AAA and PBC algorithms implemented in Eclipse™ TPS**.

**Figure 3:**
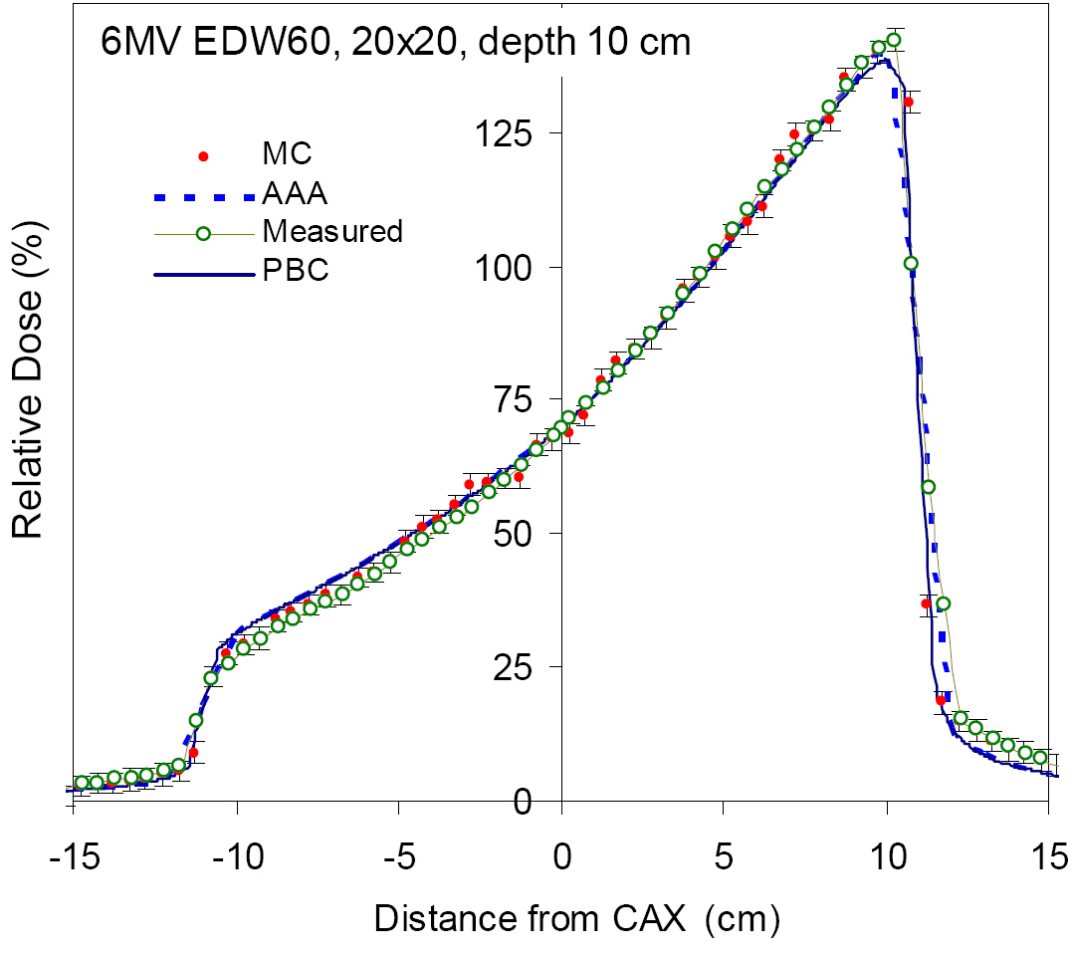
**Dose profile of a 20 × 20 cm^2 ^field at a depth of 10 cm in water for a 60° enhanced dynamic wedge measured with ionisation chamber array (Measured), calculated by Monte Carlo method (MC), as well as AAA and PBC algorithms implemented in Eclipse™ TPS**.

Figures 2 and 3 demonstrate agreement of the three calculation algorithms with the dose measurement in water for 10 × 10 and 20 × 20 cm^2 ^EDW fields at 10 cm depth. All algorithms show good overall agreement with the measurement data, however MC agrees with the measurement slightly better, especially in the out-of-field regions. Of all algorithms considered, MC has the best agreement with the 10 × 10 cm^2 ^measured data, and the agreement with 20 × 20 cm^2 ^field is excellent: the measurement points essentially overlap with MC data. Error bars on MC points demonstrate their calculated standard deviation of 1%, and most measurements fall within this range.

### Dose Calculation Algorithm Effects on Whole Breast Irradiation

Table 2 summarizes the mean, standard deviations and ranges of the target and normal tissue statistics recorded from the three WBI plans. The volumes of the DEV and PTV receiving 95% of the prescription dose using PBC calculations were not significantly different than AAA calculations (all p > 0.127). The ipsilateral whole breast volume receiving 10% of the prescription dose in the PBC plan was 10.5% higher than the AAA dose (p = 0.004). There were no statistically significant differences between PBC and AAA, or AAA and MC calculations for target or normal tissue structures. This was also true when PBC and MC calculations were compared, with the exception that the ipsilateral breast dose was 11.8% lower than the PBC calculations with MC calculations (p = 0.004).

**Table 2:** Mean, standard deviation and ranges of volumetric coverage and percent dose delivered to selected target and normal tissues as defined in Table 1 for three dose calculation algorithms during whole breast tangent radiation therapy.

	DEV [%]	PTV [%]	IPS-BR [%]	IPS-LUNG [%]	HEART [%]	CON-LUNG [%]	CON-BR [%]
PBC	97.4 (4.3)	80.3 (8.7)	67.1 (5.9)	15.1 (6.3)	12.8 (15.9)	1.1 (0.9)	18.6 (29.7)
	87.4-100.0	60.7-93.7	60.8-76.6	7.1-26.1	1.6-47.0	0.0-2.6	1.4-91.6
AAA	92.5 (8.5)	73.4 (9.3)	56.6 (7.9)	21.2 (7.6)	12.6 (16.0)	1.0 (0.6)	23.7 (25.0)
	72.0-100.0	60.6-91.3	41.0-69.1	10.9-33.6	1.3-47.0	0.8-2.2	3.0-101.1
MC	94.4 (5.5)	75.9 (13.7)	55.3 (9.4)	19.9 (6.2)	12.4 (15.6)	1.1 (0.5)	19.3 (26.8)
	83.9-100.0	60.5-98.4	42.6-69.3	10.6-31.1	1.3-44.3	0.5-2.0	5.6-94.1

### Dose Calculation Algorithm Effects on Accelerated Partial Breast Irradiation

Table 3 summarizes the mean, standard deviations and ranges of the target and normal tissue statistics recorded from the three APBI plans. The dosimetric statistics from PBC and AAA plans were not significantly different for the PTV, ipsilateral breast, heart, ipsilateral lung, and contra-lateral lung. Although not significant, the maximum dose to the contra-lateral breast was 1.9% higher for AAA compared to PBC (p = 0.030) and the average volume to the DEV receiving 95% of the prescription dose was 2.1% higher with PBC calculations compared to AAA (p = 0.012). There were no statistically significant differences between PBC and MC (p > 0.019), or AAA and MC (p = 0.100) calculations for target or normal tissue structures.

**Table 3:** Mean, standard deviation and ranges of volumetric coverage and percent dose delivered to selected target and normal tissues as defined in Table 1 for three dose calculation algorithms during partial breast radiation therapy.

	DEV [%]	PTV [%]	IPS-BR [%]	IPS-LUNG [%]	HEART [%]	CON-LUNG [%]	CON-BR [%]
PBC	99.9 (0.2)	86.1 (9.1)	32.2 (24.9)	7.5 (4.8)	3.1 (3.0)	0.3 (0.3)	2.0 (1.3)
	99.4-100.0	61.8-94.2	18.0-101.7	2.3-16.9	0.9-9.1	0.1-0.8	0.3-3.8
AAA	97.8 (2.1)	78.5 (12.2)	31.4 (21.4)	9.5 (6.1)	3.1 (2.9)	0.4 (0.3)	3.9 (2.3)
	92.5-100.0	61.5-96.1	18.0-99.6	2.3-21.4	0.8-9.1	0.0-1.0	0.3-7.4
MC	97.3 (2.9)	79.6 (12.1)	22.9 (4.5)	10.8 (5.6)	3.6 (4.4)	0.4 (0.2)	2.6 (1.3)
	91.5-100.0	61.8-96.1	14.9-28.4	2.8-20.9	0.8-12.3	0.2-0.7	0.8-4.6

### Accelerated Partial Breast versus Whole Breast Irradiation

**Figure 4:**
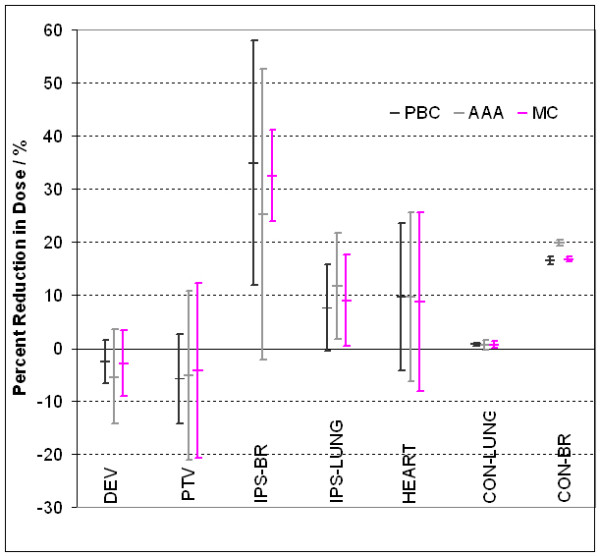
**Reductions in dose to target and normal tissue when the WBI technique is converted to ABPI**. As expected, the APBI reduces the dose to important tissues such as the ipsilateral breast, contralateral breast, heart. Note however, that the magnitude of dose reductions depends the type of dose calculation algorithm.

Table 4 summarizes the differences in volumes and doses to the target and normal tissues when comparing WBI with APBI plans for the three different algorithms. Figure 4 illustrates the difference in dose to target and normal tissues when comparing WBI with APBI for the three different algorithms. When switching from WBI to APBI with PBC, there was significant reduction in dose to the ipsilateral breast (p = 0.002). When switching from WBI to APBI with AAA, there was significant reduction in dose to the ipsilateral lung (p = 0.001). When switching from WBI to APBI with MC, there was significant reduction in dose to the ipsilateral breast and lung and contra-lateral lung (p = 0.003, p < 0.001, p = 0.001 respectively). The magnitude of the difference in dose to these structures depends on the dose calculation algorithm used.

**Table 4:** Differences in percentage of volumetric coverage and percent dose delivered to selected target and normal tissues as defined in Table 1 when WBI plans are replanned with ABPI.

	DEV	PTV	IPS-BR	IPS-LUNG	HEART	CON-LUNG	CON-BR
Dose PBC [%]	-2.5	-5.8	***35.0***	7.6	9.6	0.7	0.7
Dose AAA [%]	-5.4	-5.1	25.2	***11.7***	9.6	***0.8***	0.6
Dose MC [%]	-2.9	-4.2	***32.5***	***9.0***	8.8	***0.8***	0.7

A negative value indicates that the partial breast plan result is lower than the whole breast result. Values in italics denote significant differences between WBI and APBI doses (p < 0.006)

## Discussion

This study demonstrates very good agreement between the AAA and PBC algorithms when planning either WBI or ABPI. This suggests that there are no major concerns associated with target and normal tissue coverage if switching from PBC to AAA for WBI or ABPI. Given that AAA provides a significant improvement over the PBC plus Batho-heterogeneity corrections in lung tissue, our clinical practice has migrated from PBC to AAA along with dose calculations for the APBI clinical trial.

For APBI plans, the dose to target and normal tissue volumes varied with the dose calculation algorithm. This result is in agreement with work that explored the impact of PBC, AAA, and MC algorithms in non-clinical scenarios [11]. The volumes of the DEV and PTV receiving 95% of the prescription dose from PBC plans were higher or equal to the plans recomputed with AAA and MC. This is predictable because the lung-tissue interface is poorly calculated with PBC. If APBI plans are switched from PBC to AAA calculations, the dose to the PTV and DEV requires re-evaluation. Based on our results, a plan generated using AAA compared to PBC calculations would deliver approximately 2% more dose within the DEV. This may not have any measurable effect on tumour control but could influence the risk of late breast fibrosis because during APBI the dose per fraction is already high. This may be a particular risk if the DEV or PTV is large. The doses (and volumes receiving a specific dose) to normal structures will also correspondingly increase. Apart from the contra-lateral breast, the treatment plan can be re-configured to ensure that normal tissue constraints are maintained. This is not difficult to achieve since the doses to normal tissues are relatively independent of the calculation algorithm, with the important exception of the contra-lateral breast.

There may be a small but important difference in the contra-lateral breast dose when comparing APBI plans computed with PBC, AAA and MC algorithms. The dose to the contra-lateral breast was 2-3% higher with AAA as compared to MC. Despite the fact that dose calculation algorithms are not generally validated for dose points far away from the treatment volume and that this metric is sensitive and unstable, existing accelerated partial breast clinical trials use a maximum point dose as a constraint to the contra-lateral breast. The selection of this constraint stems from a desire to have simple planning objectives and constraints for dosimetrists. The ASTRO consensus document states that no point in the contra-lateral breast volume should receive > 3% of the prescribed dose. This work suggests that switching from the PBC to the AAA treatment planning algorithm could affect the apparent eligibility of patients for accelerated partial breast treatment. Out of ten patients in the current study, two would have failed the ASTRO contralateral breast dosimetry guideline when calculated using the PBC or MC algorithm. However, delivering an identical amount of MUs and using the same beam angles and weightings but calculated with AAA, seven patients would have not met the contra-lateral breast constraint. If reproduced across the population of patients considered for APBI, this could represent a significant reduction in eligibility. An examination of the DVH data for APBI plans suggests that relaxing the contra-lateral breast maximum dose constraint from 3% to 5% would retain eligibility for APBI without any real increase in the risk of radiation exposure or second breast cancer that is considered acceptable using existing PBC planning algorithms.

**Figure 5:**
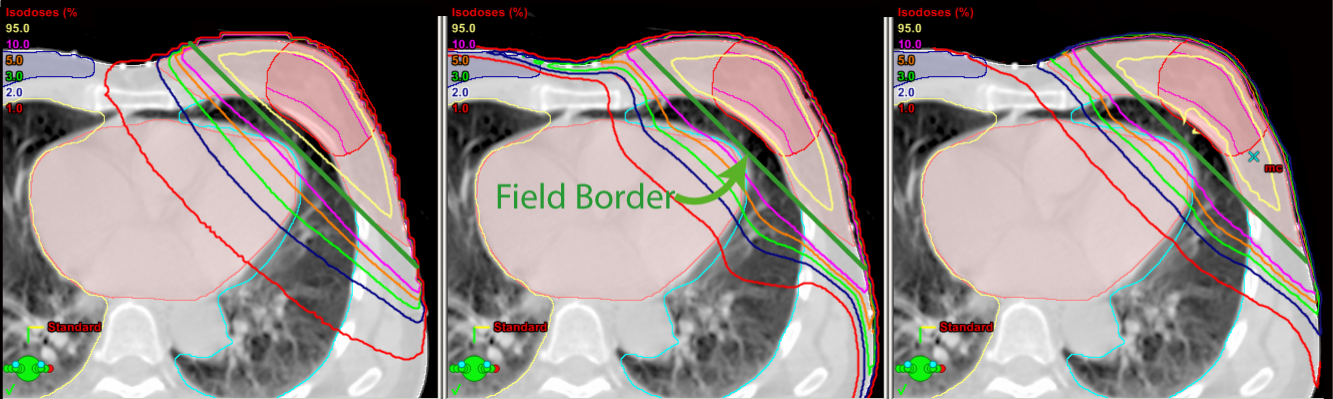
**Isodose displays of the pencil beam convolution (left), analytic anisotropy algorithm (middle) and Monte Carlo (right) for an external beam partial breast irradiation treatment**. The field border is shown in green on each of the slices. Differences in the distributions present predominantly at the lower doses. In-patient scattering modelled by the analytic anisotropic algorithm agrees well with the Monte Carlo calculations, but over predicts at the patient surface, increasing the dose to the contra-lateral breast shown in blue.

A more detailed investigation on these differences was conducted to understand where these differences stem from. Figure 5 displays three dose distributions highlighting the differences between the algorithms for tissues far from the treated volume. For PBC, the isodoses are fairly parallel to the field borders, suggesting that the in-patient scatter contributes most to the peripheral dose. For AAA, this is partially true with the exception of the dose in lung tissue and the surface of the patient, far from the field borders. This suggests that the head-scatter modelling contributes the most for tissues on the surface such as the contra-lateral breast, and in-phantom scatter contributes the most for deeper tissues. With the exception of the dose in lung, the Monte Carlo isodoses agree well with PBC for isodoses higher than 5%, and with AAA for isodoses lower than 3%.

The APBI technique often employs wedges to achieve tumor coverage, hence the accuracy of the dose calculation to the contra-lateral breast can be largely affected by the algorithm's ability to correctly calculate the in-field and penumbra dose for the EDW fields. The AAA algorithm uses a semi-analytic model to account for leakage radiation, jaw and multi-leaf transmission for open and wedged fields and can over-estimate the dose in penumbra by 1-2% when compared with MC [10]. In our centre, in-field open and wedged field agreement between measurement and calculations was better than 2% for AAA, and better than 1.5% for MC. This leads us to hypothesise that the dose differences in the contra-lateral breast are mostly due to head scatter and leakage modelling within AAA [25]. These contributions are modelled as extra-focal and electron contamination parameters within the treatment planning system, which are optimized in the beam fitting procedure. In the fitting procedure, these extra-focal parameters cannot be distinguished from other parameters in the beam tuning, leading to excellent agreement in the open field and penumbra, but not necessarily far from the open beam.

## Conclusions

There is very good agreement between PBC, AAA and MC for most tissues when treating with APBI. However, if calculation algorithms are switched from a simple pencil beam to a scatter-correction convolution/superposition algorithm, careful consideration should be given to tissues peripheral to the treated volume. In this study, it was found that a commonly used dosimetry constraint, as recommended by the ASTRO consensus document, that no point in the contra-lateral breast volume should receive >3% of the prescribed dose needs to be relaxed to >5%.

## Competing interests

The authors declare that they have no competing interests.

## Authors' contributions

PSB calculated patient plans within the treatment planning system, performed the statistical analysis, provided the initial draft and coordinated subsequent drafts of the manuscript. SZ performed the Monte Carlo calculations and helped draft the manuscript. TB assisted in the design of the study and helped draft the manuscript. IO assisted in the design of the study and helped draft the manuscript. WB assisted in the design of the study and helped draft the manuscript. All authors read and approved the final manuscript.
